# Does BCA3 Play a Role in the HIV-1 Replication Cycle?

**DOI:** 10.3390/v10040212

**Published:** 2018-04-20

**Authors:** Michaela Rumlová, Ivana Křížová, Jaroslav Zelenka, Jan Weber, Tomáš Ruml

**Affiliations:** 1 Department of Biotechnology, University of Chemistry and Technology, Technická 5, 16628 Prague, Czech Republic; ivana.krizova@vscht.cz; 2 Department of Biochemistry and Microbiology, University of Chemistry and Technology, Technická 3, 16628 Prague, Czech Republic; zelenkaa@vscht.cz; 3 Institute of Organic Chemistry and Biochemistry, Academy of Sciences of the Czech Republic, Flemingovo nám 2, 16610 Prague, Czech Republic; jan.weber@uochb.cas.cz

**Keywords:** HIV-1, BCA3, AKIP-1, M-PMV, virus incorporation, PKAc

## Abstract

The cellular role of breast carcinoma-associated protein (BCA3), also known as A-kinase-interacting protein 1 (AKIP-1), is not fully understood. Recently, we reported that full-length, but not C-terminally truncated, BCA3 is incorporated into virions of Mason-Pfizer monkey virus, and that BCA3 enhances HIV-1 protease-induced apoptosis. In the present study, we report that BCA3 is associated with purified and subtilisin-treated HIV particles. Using a combination of immune-based methods and confocal microscopy, we show that the C-terminus of BCA3 is required for packaging into HIV-1 particles. However, we were unable to identify an HIV-1 binding domain for BCA3, and we did not observe any effect of incorporated BCA3 on HIV-1 infectivity. Interestingly, the BCA3 C-terminus was previously identified as a binding site for the catalytic subunit of protein kinase A (PKAc), a cellular protein that is specifically packaged into HIV-1 particles. Based on our analysis of PKAc–BCA3 interactions, we suggest that BCA3 incorporation into HIV-1 particles is mediated by its ability to interact with PKAc.

## 1. Introduction

HIV-1 has a limited genome, which leverages alternative splicing to produce nine products: three polyprotein precursors comprising structural (Gag), enzymatic (Pol) and envelope (Env) proteins, and the six accessory proteins Tat, Rev, Vif, Vpr, Nef and Vpu. Therefore, it is not surprising that HIV-1 depends on a large number of cellular proteins and protein complexes for replication. In addition, many cellular proteins have been found inside or on the surface of the virus. These cellular proteins are incorporated into HIV particles during assembly and budding, and they often play important roles during the early phase of viral infection. However, some proteins become incorporated into the virion due to their proximity during assembly/budding or due to their ability to bind a “real” HIV-1 interaction partner.

The catalytic subunit of cAMP-dependent protein kinase A (PKAc) is among the cellular proteins packaged within highly purified HIV-1 particles [1]. HIV-1-associated PKAc is active and able to phosphorylate substrates. Some evidence indicates that HIV-1-associated active PKAc plays an important role during post-entry events comprising the early stage of reverse transcription [2].

Breast cancer-associated protein 3 (BCA3) was first identified in breast cancer cell lines [3]. Sastri et al. identified the same protein as a binding partner for the catalytic subunit of PKAc and named it A-kinase-interacting protein 1 (AKIP-1) [4]. Since then, several laboratories have studied the cellular role of BCA3, but their findings have been somewhat contradictory. While some researchers showed that BCA3 modulates NF-κB and PKAc activity resulting in induction of apoptosis and reduction of tumor progression [4,5,6,7], others reported BCA3-dependent stimulation of tumor growth and angiogenesis [2,8]. Work by Booij et al. indicated that BCA3 does not regulate the activity of NF-κB at all [9]. Some recent papers suggest that BCA3 is upregulated in cardiomyocytes in response to oxidative stress to reduce cardiomyocyte apoptosis in vitro [10] and myocardial ischemia/reperfusion injury in BCA3-overexpressing transgenic mice [9]. The latter study showed that the cardioprotective effects of BCA3 are due to its interaction with mitochondrial ATP synthase, resulting in permeability transition pore (MPT) stabilization [9]. These discrepancies in the cellular function of BCA3 could be explained by its different roles in different cell lines and/or tissues.

We identified BCA3 during a yeast two-hybrid screen for binding partners of Mason-Pfizer monkey virus (M-PMV) protease [11]. We found that full-length BCA3, but not a C-terminally truncated form, is incorporated into M-PMV virions. Next, we showed that BCA3 could also interact with HIV-1 protease (PR) and accelerate p53 transcriptional activity leading to elevated apoptosis [12].

Here, we studied the potential role of BCA3 in the HIV-1 replication cycle. Regardless of the level of BCA3 expression, we found that the protein associated with purified and subtilisin-treated HIV-1 particles. By progressively deleting individual BCA3 exons, we found that the BCA3 C-terminal region corresponding to amino acids 165–210 of exon 6 is responsible for its incorporation into HIV-1. To analyze the BCA3 binding site in HIV-1, we applied a similar “domain-deletion” approach to HIV-1, and prepared a series of vectors with progressive truncation of the Gag and Pol regions. Immunodetection of the presence of BCA3 in released HIV-1 particles assembled from these truncated polyproteins did not reveal an HIV-1 part/domain/motif responsible for BCA3 binding. However, we established the C-terminus of BCA3 as the domain responsible for the previously published interaction of BCA3 with PKAc [13]. As only BCA3 containing the C-terminal PKAc-binding domain is associated with HIV-1, we can hypothesize that BCA3 is co-packaged into HIV-1 particles due to its ability to interact with PKAc.

## 2. Materials and Methods

### 2.1. Plasmid Construction

All DNA manipulations were carried out using standard subcloning techniques, and plasmids were propagated in *E. coli* DH5α. All newly created constructs were verified by DNA sequencing. The full-length gene encoding human BCA3 was obtained as previously described [12]. Truncated forms of BCA3 were prepared by ligating two PCR fragments encoding particular parts of the BCA3 gene into pCMV-HA (Clontech, Fremont, CA, USA), resulting in HA-Δ2BCA3, HA-Δ3BCA3, HA-Δ4BCA3, HA-Δ5BCA3 and HA-Δ6BCA3. The psPAX2 vector, a lentiviral packaging vector that encodes HIV-1 *gag* and *pol* regions with deletion of the *env* gene, was kindly provided by Dr. J. Luban. To prepare HIV vectors with deletions in the *pol* gene, we used standard subcloning techniques to introduce PCR fragments encoding particular parts of the *pol* gene into a previously described HIV-1 helper vector (HIV-1 SphI-SbfI fragment (nts 2433–3828) in pUC19) [14]. The HIV-1 Gag construct was prepared by ligation of PCR fragment encoding HIV-1 *gag* gene into pCMV. To introduce deletions within the *gag* gene, a combination of three previously published vectors was used: HIV-1 helper vector, ΔMA-CA-NC-SP2pET22b vector with deletion of the sequence encoding amino acids 16–99 within MA [15], and the HIV-1/CREB chimeric vector GagΔ10CREB DLZ, in which the NC domain is replaced with a CREB leucine zipper domain [14]. Vectors for expression of CA, tat and rev were prepared by subcloning the corresponding PCR regions into an HA- or c-myc-tagged pCMV vector. Point mutation D25N in the *pro* gene was introduced by two-step PCR mutagenesis using primers carrying the desired mutations and suitable restriction sites. Further details of the cloning strategy and full sequences of all PCR primers can be obtained from the authors upon request. Plasmid pNL4-3 was obtained through the NIH AIDS Reagent Program, Division of AIDS, National Institute of Allergy and Infectious Diseases (NIAID), National Institutes of Health (NIH) from Malcolm Martin.

### 2.2. Cell Lines and Protein Expression

HEK-293 cells were grown in Dulbecco’s Modified Eagle’s Medium (DMEM, Sigma, s.r.o., Prague, Czech Republic) supplemented with 10% fetal bovine serum (Sigma) and 1% L-glutamine (Sigma) at 37 °C under 5% CO_2_. Typically, cells were plated at a density of 3 × 10^5^ cells/mL one day before transfection. The following day, cells were transfected with the appropriate plasmid(s) using polyethylenimine (PEI, 1 mg/mL) at a 2:1 PEI:DNA ratio or X-tremeGENE HP DNA Transfection Reagent (Roche, s.r.o., Prague, Czech Republic) according to the manufacturer’s instructions. The cells were grown for 24–48 h post-transfection. Further processing depended on the type of experiment. Two clones (D1, D2) of stably transfected HEK-293 cells expressing HA-BCA3 were prepared as described elsewhere [11]. TZM-bl cells were obtained through the NIH AIDS Reagent Program, Division of AIDS, NIAID, NIH from John C. Kappes, Xiaoyun Wu and Tranzyme Inc. (Durham, NC, USA), and maintained in DMEM.

### 2.3. HIV-1 Purification and Subtilisin Treatment

Viral particles were prepared according to a method adapted from that of Ott et al. [16]. Briefly, 48 h post-transfection, virus-containing culture media were centrifuged at 1000× *g* for 5 min and filtered through a 0.45 µm filter. Virions were isolated by ultracentrifugation through a 20% sucrose cushion at 40,000 rpm for 1 h in a SW41 rotor, and the pellet was resuspended in 100 µL PBS. A 90 µL aliquot of concentrated virus was purified using a sucrose density gradient. Individual fractions were analyzed by Western blot using rabbit anti-HIV-1 CA and mouse anti-HA antibodies. A 10 µL aliquot of the concentrated virus suspension was added to an equal volume of subtilisin digestion buffer (2 mg/mL subtilisin, 40 mM Tris-HCl pH 8.0, 2 mM CaCl_2_) in a 1:1 ratio in the presence or absence of 1% Triton X-100. The mixture was incubated at 37 °C overnight, and the digested samples were analyzed immunochemically by Western blot.

### 2.4. TZMb1 Assay

Forty-eight h post transfection, HIV-1 (NL4-3)-containing culture medium was centrifuged at 3000 rpm for 5 min to remove cells and cell debris. Cleared supernatants were used for viral titration in TZMbl cells. Here, 30,000 TZMbl cells per well were infected in 10-fold dilution format in triplicate. After 2 days, firefly luminescence was measured using Steady-Glo luminescence assay system (Promega, Madison, WI, USA) with Victor X3 plate reader (Perkin Elmer, s.r.o., Prague, Czech Republic), and virus infectivity was determined.

### 2.5. Western Blotting and Antibodies

Proteins were resolved by SDS–PAGE and blotted onto a nitrocellulose membrane. The membrane was incubated with primary antibody overnight and then incubated with HRP-conjugated secondary antibody for 1–2 h at 4 °C. The antigen–antibody complexes were detected by addition of West Femto Chemiluminescent Substrate (Thermo Scientific, s.r.o., Prague, Czech Republic) and visualized using a FUSION 7S system (Vilber Lourmat, Marne-la-Vallée, France). The following primary antibodies were used: monoclonal anti-HA peroxidase conjugate clone HA-7 and anti-alpha-tubulin (B512) antibodies from Sigma-Aldrich, anti-PKA(C) antibody from BD Pharmingen, and rabbit anti-HIV-1 CA antibody produced in-house. All secondary HRP-conjugated antibodies (anti-rabbit IgG, anti-mouse IgG) were purchased from Sigma-Aldrich.

### 2.6. Co-Immunoprecipitation

Co-immunoprecipitation was carried out as previously described [12,17]. Briefly, HEK-293 cells were grown on 100 mm plates and transfected with HA-BCA3 or HA-Δ6BCA3. At 48 h post-transfection, the cells were washed with PBS and lysed in 500 µL CO-IP buffer B (20 mM Tris-HCl, pH 7.5, 150 mM NaCl, 1 mM EDTA, 1 mM DTT, 1 mM MgCl_2_, 0.5% NP-40) containing Halt Protease Inhibitor Mix (Thermo Scientific) for 30 min on ice. One-fifth of the total cell lysate sample was used for Western blot analysis. The rest of the cell lysate was cleared by centrifugation and diluted with 1 mL CO-IP A buffer (20 mM Tris-HCl, pH 7.5, 150 mM NaCl, 1 mM EDTA, 1 mM DTT, 0.1 mM MgCl_2_). The primary antibody, monoclonal anti-PKA(C) or anti-HA, was added to the cell extract and incubated overnight at 4 °C. The next day, 20 µL Protein-A/G-Sepharose beads were added, and after 2 h incubation at 4 °C, the immunocomplexes were collected by centrifugation. The pellets were washed with CO-IP A buffer and PBS, and immunoprecipitated proteins were separated by SDS–PAGE, transferred onto a nitrocellulose membrane, and detected by Western blot analysis.

### 2.7. siRNA Knockdown

siRNAs against BCA3 (C11orf17) were purchased from Ambion (Thermo Fisher Scientific, s.r.o., Prague, Czech Republic). One day before transfection, HEK 293 cells were seeded in 12-well plate at the density 3 × 10^5^ cells/mL. Next day the cells were transfected with siRNA at various concentrations using siPORT (Ambion), according to the manufacturer’s instructions. At 24 h post the first transfection, the cells were transfected with *HA-BCA3* expression vector (0.4 µg per well) using Fugene. At 24 h post-second-transfection, the cells were harvested and analyzed for presence of HA-BCA3 by Western blot analysis. In an experiment when the effect of BCA3 knockdown on HIV-1 release was analyzed, HEK 293 cells were grown in 60 mm plates and the transfection procedure was carried out as described above, with the exception that the *psPAX2* vector was cotransfected with that encoding HA-BCA3. At 24 h post-second-transfection, the culture media were filtered through 0.45 µm filter and ultracentrifuged through a 20% sucrose cushion at 40,000 rpm for 1 h in a SW41 rotor. Both the viral pellet and producing cells were analyzed by Western blot and quantification of virus release was determined as a ratio of protein bands’ intensity of virion-associated CA to cell-associated CA by using Fusion CAPT Advance software (Vilber Lourmat, Marne-la-Vallée, France).

### 2.8. Fluorescence Microscopy

Fluorescence microscopy was carried out as previously described [12]. Briefly, HEK-293 or HeLa cells were plated on a glass coverslip at a density of 1 × 10^5^ cells/mL, placed in a 6-well plate, and incubated in DMEM supplemented with 10% FBS (Sigma) and 1% L-glutamine (PAA) at 37 °C and 5% CO_2_. The next day, the cells were transfected with appropriate plasmid DNA using FuGENE HD Transfection Reagent (Roche) according to the manufacturer’s protocol. At 48 h post-transfection, the cells were washed with PBS, fixed with 4% formaldehyde, and incubated for 30 min at room temperature. The cells were then washed with PBS and incubated for 10 min in 0.4% Triton X-100, followed by 2 × 5 min incubations with 0.1% Triton X-100. The cells were then incubated with primary fluorescently labeled antibodies for 60 min at room temperature in the dark, washed twice with PBS, and mounted using mounting media (Vectashield, Vector Laboratories, INC., Burlingame, CA, USA). The samples were then analyzed using a laser scanning confocal microscope (Leica, Pragolab s.r.o, Prague, Czech Republic) or an Olympus IX 81 microscope. Image analysis was performed with Imaris software (Bitplane, Zurich, Switzerland).

## 3. Results

### 3.1. BCA3 Is Incorporated into HIV-1 Particles

**Figure 1:**
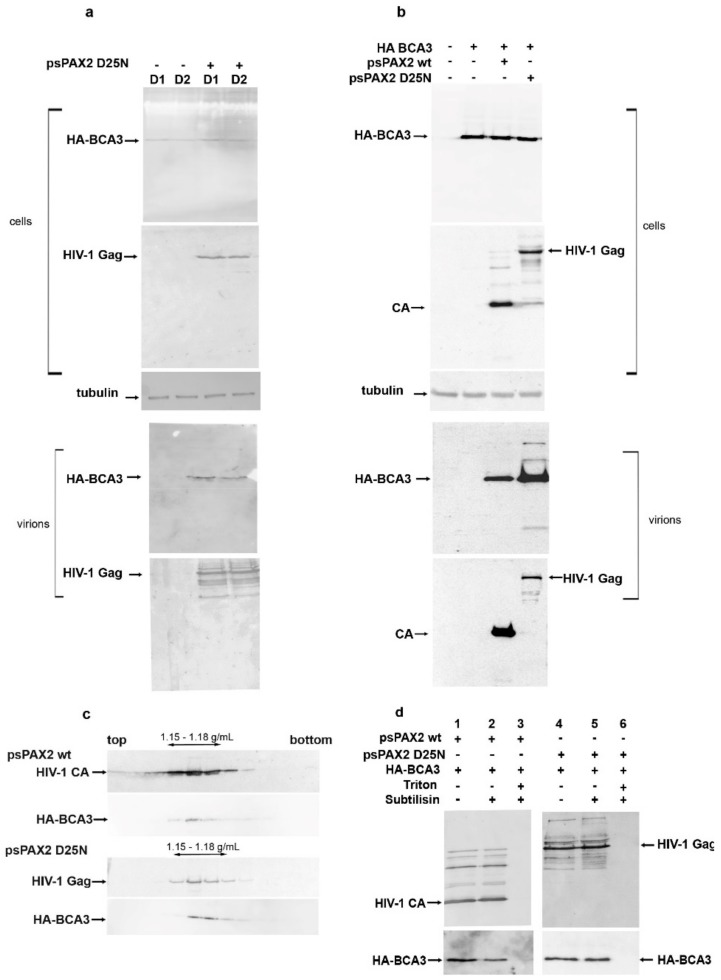
Presence of BCA3 in HIV-1 particles. (**a**) Clones D1 and D2 of HEK 293 cells stably expressing a low level of HA-BCA3 were transfected with a vector encoding HIV Gag and Pol, with inactive protease (*psPAX2 D25N*). At 48 h post-transfection, the virions released into culture media were isolated by ultracentrifugation through a 20% sucrose layer. The producing cells and the viral pellet were analyzed by Western blotting; (**b**) HEK 293 cells were cotransfected with vectors encoding the HIV Gag–Pol region with either active (*psPAX2*) or inactive (*psPAXD25N*) protease and *HA-BCA3* vector. The virions were concentrated, and the presence of BCA3 in transfected cells and isolated virions was analyzed immunochemically; (**c**) Virions concentrated from cells transfected with *psPAX2* + *HA-BCA3* or *psPAXD25N* + *HA-BCA3* were then loaded onto a linear (20–65%) sucrose gradient and centrifuged to equilibrium. Individual fractions were analyzed by Western blot; (**d**) Virions released from cells transfected with *psPAX2 + BCA3* or *psPAXD25N + BCA3* were separated by ultracentrifugation through a sucrose layer and transferred into subtilisin-containing cleavage buffer, optionally in the presence of Triton X-100% (lanes 3, 6). The presence of BCA3 inside intact HIV-1 particles was detected immunochemically. Monoclonal anti-HA antibody and rabbit anti-HIV-1 CA were used to detect HA-BCA3- and HIV-1 CA-related proteins, respectively.

Previously, we showed that full-length but not C-terminally truncated BCA3 is incorporated into M-PMV [11] and slightly increases its infectivity. We also found that BCA3 facilitates HIV-1 PR-mediated apoptosis [12]. To analyze its putative role in the HIV-1 replication cycle, we first assessed whether BCA3 is incorporated into HIV-1 particles. To avoid possible overexpression of BCA3, we used stably transfected cell lines in addition to transiently transfected ones. Two clones (D1, D2) of a HEK 293 cell line stably expressing a low level of HA-tagged BCA3 were prepared as previously reported [11]. As BCA3 co-localizes with HIV-1 PR, we used both active and inactive (D25N) forms of HIV-1 PR to determine whether BCA3 is cleaved by HIV-1 PR. HIV-1 expression vectors encoding HIV-1 Gag and Pol with active (*psPAX2*) or inactive (p*sPAX2D25N*) PR were transfected either into the D1 and D2 cell lines or HEK 293 cells, alone or together with a vector encoding HA-BCA3. After two days, HIV-1 particles were collected from the culture media. The presence of BCA3 in virus released from the D1 and D2 cell lines (Figure 1a) and HEK 293 cells (Figure 1b) was analyzed immunochemically by Western blotting. BCA3 protein was detected in released HIV-1 particles, similar to our previous findings for M-PMV.

HIV-1 particles released from HEK 293 cells were loaded onto a 20–65% sucrose density gradient and ultracentrifuged overnight to equilibrium. Analysis of individual gradient fractions revealed the presence of BCA3 and HIV-1 virions in fractions corresponding to a sucrose density of 1.15–1.18 g/mL (Figure 1c). To verify that BCA3 is associated with HIV-1 particles and not associated with co-pelleted cellular microvesicles or nonspecifically adsorbed to the virus surface, we carried out subtilisin treatment [16]. The HIV-1 VLPs isolated from cells cotransfected with *HA-BCA3* were digested with subtilisin in the presence or absence of detergent (Figure 1d). While subtilisin digestion completely removes co-pelleted microvesicles and surface-displayed proteins, an HIV-1 particle enveloped by a cell-derived membrane should remain intact [16]. Thus, only virus-incorporated BCA3 should resist subtilisin treatment in the absence of detergent. In contrast, a combination of subtilisin and detergent can also degrade the proteins within the viral particles. Indeed, in the presence of Triton X-100, which disrupts the viral membrane, the protein content was completely digested by subtilisin (Figure 1d, lanes 3 and 6). In the absence of detergent, the intact HIV particles remained undigested (Figure 1d, compare lanes 2 to 3 and 5 to 6), and BCA3 association with HIV-1 particles was confirmed. These experiments also revealed that BCA3 is not cleaved by HIV-1 PR inside mature particles.

### 3.2. The BCA3 C-Terminus Is Essential for Incorporation into HIV-1

**Figure 2:**
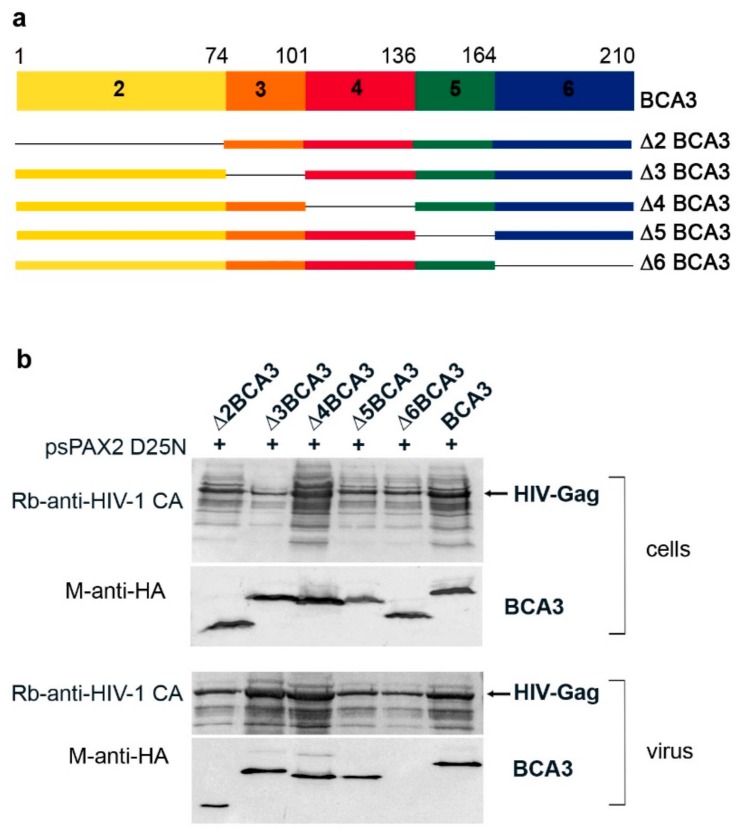
Analysis of BCA3 truncated forms. (**a**) Schematic representation of BCA3, its individual exons 2–6, and the exon-deleted BCA3 variants used in this study. (**b**) HEK-293 cells were cotransfected with *psPAX2D25N* and the vectors encoding individual truncated *BCA3* variants. At 48 h post-transfection, cultivation media were filtered through a 0.45 µm filter, and HIV-1 particles were concentrated by ultracentrifugation through a 20% sucrose cushion. Both transfected cells and purified HIV-1 particles were analyzed by Western blotting using anti-HIV-1 CA and anti-HA antibodies.

Next, we sought to identify the part of BCA3 responsible for its incorporation into HIV-1 particles. As BCA3 consists of six exons, with translation start in exon 2 [3], we prepared a panel of HA-tagged BCA3-expression vectors with gradual deletions of individual exons: Δ2BCA3, Δ3BCA3, Δ4BCA3, Δ5BCA3 and Δ6BCA3 (Figure 2a). These individual BCA3 vectors were cotransfected with *psPAX2(D25N)* into HEK 293 cells. Two days after transfection, we performed Western blot analysis of both the cell lysates and HIV-1 particles isolated from the cultivation media by centrifugation through a sucrose cushion (Figure 2b). All expected truncated forms of BCA3 were expressed in HEK 293 cells, and, with the exception of C-terminally truncated Δ6BCA3, they also were incorporated into HIV-1 particles. This result is consistent with our previous finding that Δ6BCA3 is not incorporated into M-PMV virions [11].

**Figure 3:**
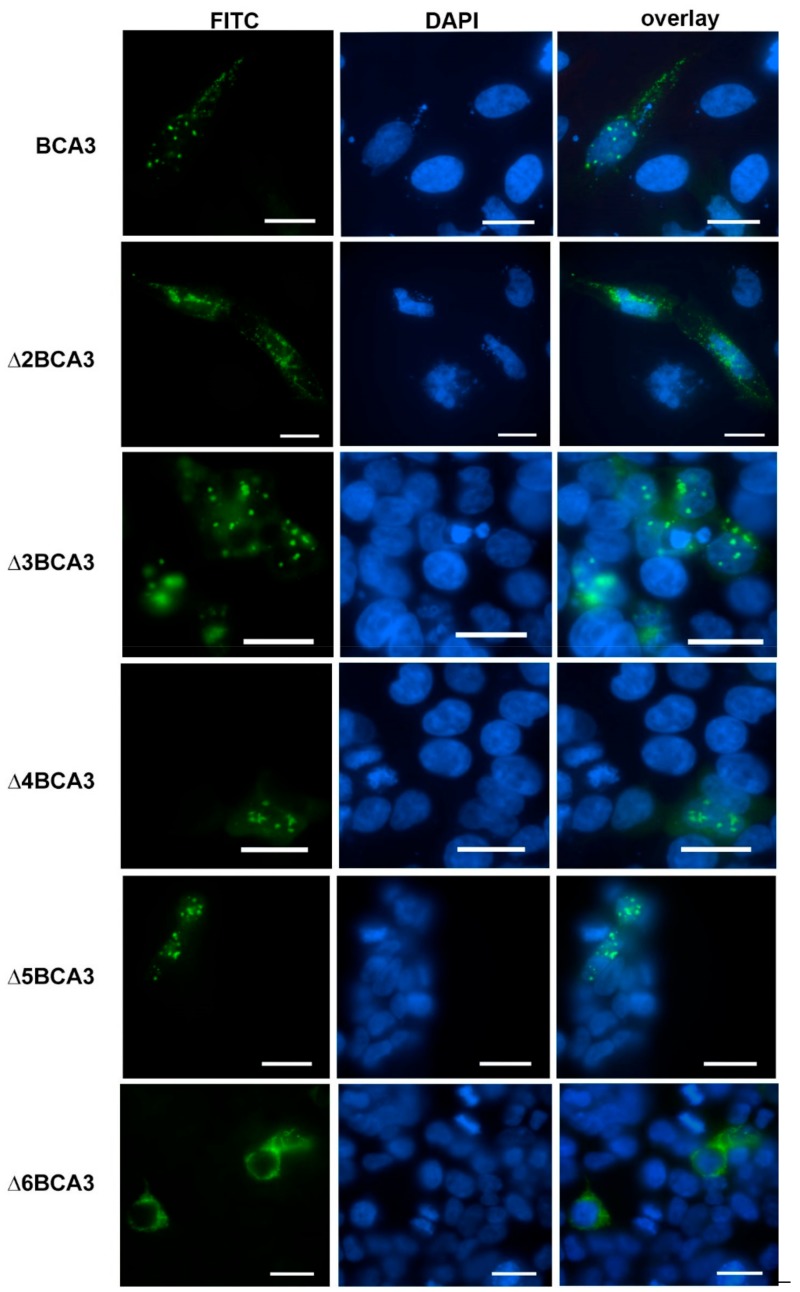
Immunolocalization of individual exon-deleted BCA3 variants in HeLa cells. The cells were transfected with vectors expressing the indicated truncated forms of BCA3. At 48 h post-transfection, the cells were stained with 4'6-diamidino-2-phenylindole (DAPI, blue) and HA-FITC (fluorescein isothiocyanate) antibody (green) and visualized using confocal microscopy. Bars represent 20 µm.

To analyze whether the inability of Δ6BCA3 to be incorporated into HIV-1 particles may be connected to a distinct cellular localization, we employed confocal fluorescence microscopy (Figure 3). HeLa cells expressing the individual truncated forms of BCA3 were stained with FITC-labeled anti-HA antibody. Full-length BCA3 and the Δ3BCA3, Δ4BCA3 and Δ5BCA3 variants were observed in both the nucleus and cytosol, with a speckle-like appearance. In contrast, Δ2BCA3 and Δ6BCA3 were found only in the cytosol, and had a distinct appearance.

**Figure 4:**
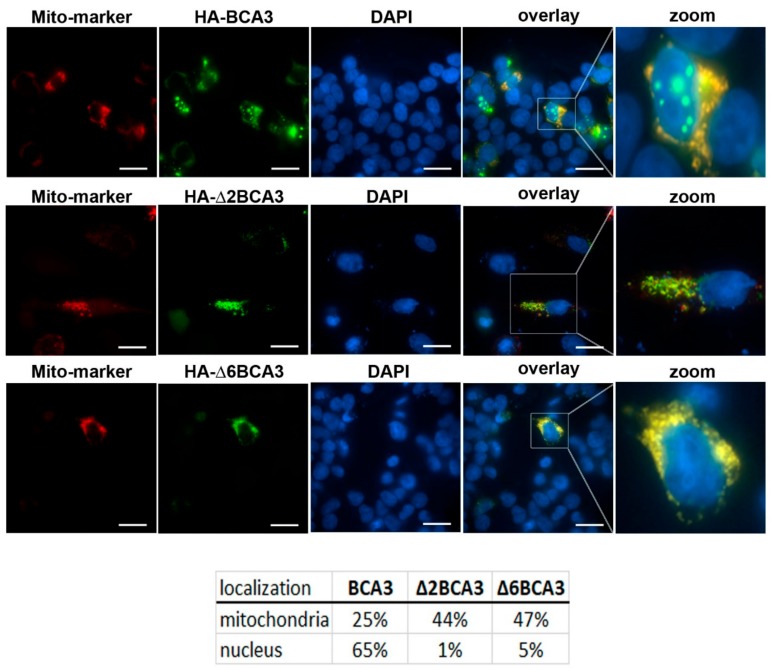
Localization of BCA3. HeLa cells were cotransfected with *BCA3* (upper panels), Δ2BCA3 (middle panels) or *Δ6BCA3* (lower panels) with mitochondrial marker Mito-DsRed. At 48 h post-transfection, the BCA3 protein was stained using monoclonal FITC-labeled anti-HA antibody and visualized using confocal microscopy. Bars represent 20 µm. The image analysis was performed with Imaris software (Bitplane).

The extranuclear localization of Δ2BCA3 is in agreement with the previous finding that exon 2 encodes a nuclear localization signal (NLS) comprising R^14^ and R^15^ [4]. Thus, it is unsurprising that deletion of the entire exon 2 (amino acids 1–74) blocked Δ2BCA3 from translocation into the nucleus. However, deletion of exon 6 does not abolish the NLS, and the extranuclear localization of Δ6BCA3 cannot be explained as easily. Because BCA3 has also been found in mitochondria [10,12,18], we co-expressed BCA3, Δ2BCA3 and Δ6BCA3 with a mitochondrial marker (Mito-DsRed, Clontech, Fremont, CA, USA). We detected full-length BCA3 in the nucleus and cytosol, where it partially co-localized with the Mito-DsRed marker (Figure 4). Δ2BCA3 was found in the cytosol and mitochondria with a dot-like appearance, and Δ6BCA3 formed dot- and thread-like structures co-localized predominantly with mitochondria (Figure 4). To verify the presence of truncated forms of BCA3 in subcellular fractions, we separated the cell lysates using two low-speed centrifugation steps and subsequently clarified the lysates from pelleted cell debris and nuclei by centrifugation at 3000× *g*, and mitochondria by centrifugation at 16,000× *g*. BCA3, Δ2BCA3 and Δ6BCA3 were identified in cytosolic and mitochondrial fractions [19]. Even though no nuclear localization was observed for Δ2BCA3 and Δ6BCA3 by confocal microscopy, pellets containing nuclei separated by low-speed centrifugation contained both Δ2BCA3 and Δ6BCA3. The dot-like appearance of Δ2BCA3 and Δ6BCA3 suggests that the proteins either aggregated or formed complexes in the cytosol, and pelleted at low-speed centrifugation. Based on these results, we were not able to determine whether Δ6BCA3 is present only in mitochondria or is also present in cytosol.

### 3.3. Domain-Deletion Analysis Does Not Reveal the HIV Domain Responsible for BCA3 Binding

**Figure 5:**
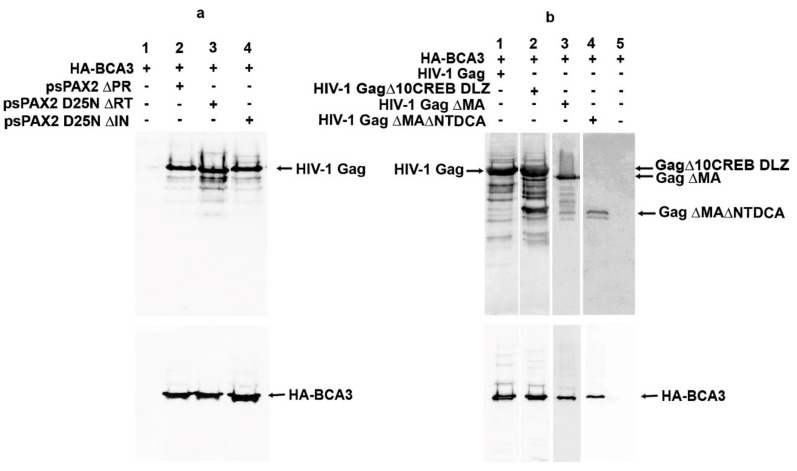
Presence of BCA3 in HIV-1-like particles with various deletions within the *pol* (**a**) and *gag* (**b**) regions. HEK-293 cells were cotransfected with indicated vectors encoding BCA3 and HIV-1 Gag–Pol with various deletions within the *pol* (**a**) and *gag* (**b**) regions. The viruses released into culture media were filtered through a 0.45 µm filter and isolated by ultracentrifugation through a 20% sucrose layer. Viral pellets were analyzed by Western blotting.

Despite the work of several laboratories, the cellular role of BCA3 remains unclear. Several features of BCA3 complicate efforts to characterize it: (i) it has no homology with any other known protein, (ii) it does not encode any known structural/functional domain, and (iii) it is expressed at a very low level in the majority of commonly used cell lines. Moreover, commercially available antibodies to detect BCA3 are often not specific. To better understand BCA3 incorporation into HIV-1, we therefore attempted to identify the part or domain of HIV-1 responsible for BCA3 incorporation. In our previous work, we identified BCA3 as an M-PMV PR interaction partner and also showed that BCA3 is associated with HIV-1 PR. Thus, we first deleted the *pro* region from *psPAX2* (*psPAX2∆PR*) and analyzed whether BCA3 is still incorporated into these modified HIV-1 particles. Surprisingly, deletion of the *pro* region did not abolish BCA3 incorporation into ∆PR HIV-1 particles (Figure 5a, lane 2), suggesting that while BCA3 can associate with active dimeric HIV-1 PR, it does not bind to the PR precursor. Similarly, deletion of the *RT* and *IN* parts of the *pol* gene did not abolish BCA3 incorporation (Figure 5a, lanes 3 and 4). Next, we prepared a series of HIV-1 variants with *gag* gene and its truncations in pCMV vector (Figure 5b). These variants included deletions in the nucleocapsid domain (NC), which was replaced with the dimerization leucine zipper (DLZ) from CREB transcription factor (GagΔ10CREB DLZ) [14]; amino acids 16–99 of MA (Gag ΔMA) [15]; and amino acids 16–99 of MA in combination with the N-terminal domain of capsid protein (NTD CA) (Gag ΔMA ΔNTDCA). However, in all these cases, BCA3 was still present within the HIV-1-like particles (Figure 5b, lanes 1–4). We next individually cloned the parts of HIV-1 essential for expression, assembly and release of the virus (i.e., CA-CTD, p6, tat and rev) into pCMV expression vectors. Immunoprecipitation analysis did not reveal any specific interactions between these viral components and BCA3 [19].

### 3.4. PKAc Interacts with BCA3, Facilitating Incorporation into HIV-1 Particles

**Figure 6:**
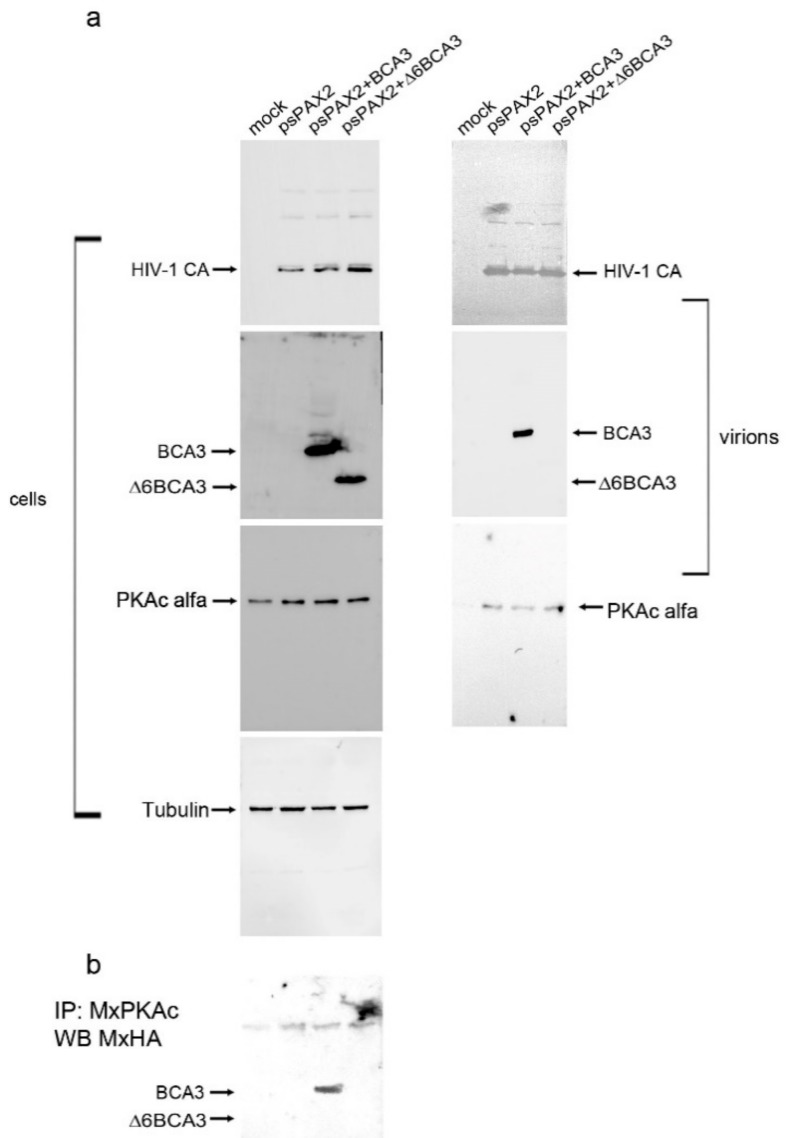
Incorporation of BCA3 and PKAc into HIV-1 particles. (**a**) HEK-293 cells were cotransfected with *psPAX2* and vectors encoding HA-tagged BCA3 (*HA-BCA3*) or its C-terminally truncated form (*HA-Δ6BCA3*). The virions released from the transfected cells were concentrated by ultracentrifugation, and cell and virus samples were analyzed by Western blotting using the antibodies indicated; (**b**) Co-immunoprecipitation of BCA3 variants with PKAc. HEK-293 cells were transfected with *HA-BCA3* or *HA-Δ6BCA3*. At 48 h post-transfection, the cells were lysed and the proteins were immunoprecipitated using anti-PKAc antibody. Precipitates were resolved by SDS–PAGE, blotted and developed using anti-HA antibody.

By searching the literature, we found evidence that the C-terminal part of BCA3, specifically amino acids 150–179 spanning the 13 C-terminal amino acids of exon 5 and 16 N-terminal amino acids of exon 6, interacts with the catalytic subunit of PKAc [13]. As the catalytic subunit of PKAc has been shown to associate with highly purified HIV-1 particles [1], we asked whether BCA3 might be incorporated into HIV-1 particles due to its interaction with endogenous PKAc. To test this hypothesis, we cotransfected HEK 293 cells with *HA-BCA*3/*psPAX2* and *HA-Δ6BCA3/psPAX2*, and 48 h post-transfection, we collected the released HIV-1 particles and isolated them by ultracentrifugation. The cell-associated (Figure 6, left panels) and virus-associated (Figure 6, right panels) proteins were analyzed by Western blotting. In agreement with published data [1,2], we detected PKAc in all HIV-1 particles released from control cells, as well as BCA3- and Δ6BCA3-expressing cells. As expected, no Δ6BCA3 was identified in the released HIV-1 particles. To verify the interaction of PKAc with the BCA3 C-terminus, the BCA3- and Δ6BCA3-expressing cells (Figure 6a, left panels) were lysed and subjected to co-immunoprecipitation followed by Western blot analysis (Figure 6b). This experiment confirmed that full-length BCA3, but not the C-terminally truncated Δ6BCA3 variant, interacts with endogenous PKAc.

### 3.5. BCA3 Incorporation Does Not Affect HIV-1 Infectivity

**Figure 7:**
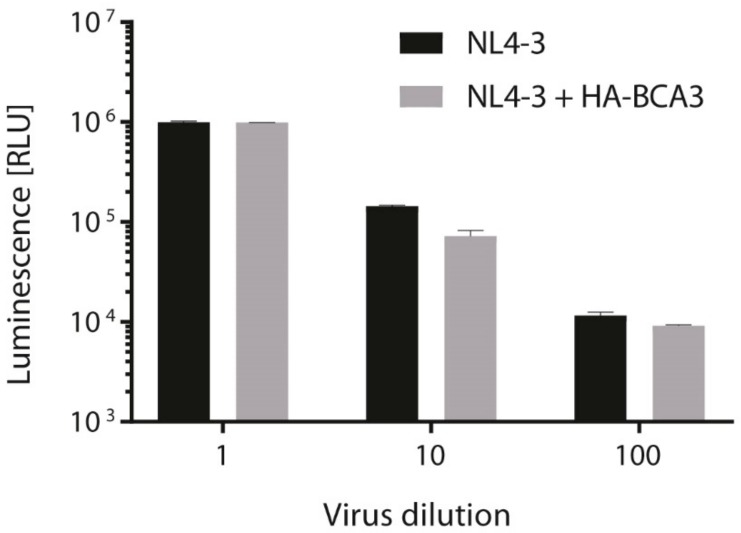
HIV infectivity determination by TZMbl assay. Determination of HIV infectivity of NL4-3 viruses prepared by transfection of HEK293T cells with pNL4-3 in the presence or absence of HA-tagged BCA3. Forty-eight h after transfection, cleared supernatants were used to infect TZMbl cells with serial 10-fold dilutions of viruses in triplicate. After 48 h post-infection, luminescence levels were measured and expressed in RLU (relative light unit). Graph shows average and standard deviation from three independent transfections.

Finally, we tested whether incorporation of BCA3 affects HIV-1 infectivity. We transfected 293T cells with the fully infectious molecular clone pNL4-3 alone or cotransfected together with HA-tagged BCA3, and 48 h post-transfection, used an aliquot of virus-containing media to infect TZMb1 cells. At 48 h post-infection, virus infectivity was quantified by measuring firefly luciferase activity. Although a small decrease of infectivity was detected for 10-fold- and 100-fold-diluted NL4-3 viruses with incorporated BCA3, there was no effect of BCA3 on the final HIV-1 titer (Figure 7).

**Figure 8:**
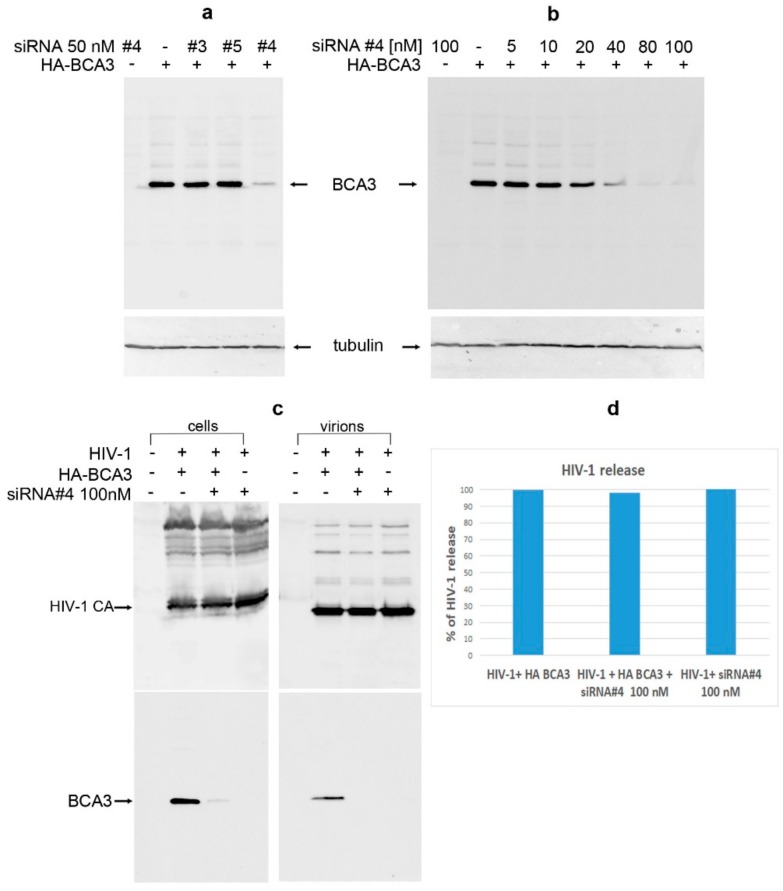
BCA3 knockdown. (**a**) siRNAs against BCA3 (#3, #4 and #5) at final concentration of 50 nM were transfected into HEK 293 cells. At 24 h post-transfection, the cells were transfected again with *HA-BCA3* expression vector. At 24 h after the second transfection, the cells were harvested and analyzed for presence of HA-BCA3 by Western blot analysis using monoclonal anti-HA antibody. Monoclonal antitubulin antibody was used as a loading control. (**b**) HEK 293 cells were first transfected with indicated amounts of siRNA #4, and 24 h later transfected with *HA-BCA3.* At 24 h after the second transfection, the cells were harvested and analyzed for presence of HA-BCA3 by Western blot. (**c**) HEK 293 cells were grown in 60 mm plates and subsequently transfected with siRNA #4 and HA-BCA3 as described above, with the exception that *psPAX2* vector was cotransfected together with *HA-BCA3*. At 24 h after second transfection, the viral particles from culture media were concentrated by ultracentrifugation, and both viral pellet and producing cells were analyzed by Western blot. (**d**) The virus-release ratio, estimated from intensity of bands corresponding to virion-associated CA versus cell-associated CA, did not reveal any significant difference when the amount of virions released from the nonsilenced cells was set as 100%.

To verify that BCA3 has no effect on HIV-1 infectivity in this system, we used siRNA against BCA3 to knock-down its expression. Three various siRNAs (#3, #4 and #5) were tested (Figure 8a), one of which designated here as number 4 (#4), at concentration 80 nM or higher, efficiently silenced the HA-BCA3 expression. Following optimization of siRNA #4 amount (Figure 8b), the final concentration of 100 nM siRNA #4 was used for further experiment. The HEK 293 cells were first transfected with siRNA #4, and 24 h later transfected with *HA-BCA3* and *psPAX2* at indicated combinations (Figure 8c). At 48 h after the first transfection, the culture media were filtered and viral particles were ultracentrifuged through sucrose cushion. Both transfected cells and viral pellets were analyzed by Western blot (Figure 8c,d). No effect on HIV-1 release was observed in BCA3-expressing cells when compared to those where BCA3 was silenced (Figure 8c,d). The virus-release ratio, estimated from intensity of bands (error bars ± 15% in two independent experiments) corresponding to virion-associated CA versus cell-associated CA, did not reveal any significant difference: When the amount of virions released from the nonsilenced cells was set as 100%, the silenced *HA-BCA3-*transfected and -nontransfected cells released virions with 96% and 102% efficiency, respectively (Figure 8c,d).

## 4. Discussion

In this work, we studied the potential role of the recently identified HIV-1 PR interaction partner BCA3 [12] in the HIV-1 replication cycle. We found that, similar to its behavior with M-PMV, BCA3 is packaged into HIV-1 particles, and the BCA3 C-terminus is responsible for this incorporation. Using a combination of co-expression and co-immunoprecipitation experiments, we were not able to identify an HIV-1 domain responsible for BCA3 binding. However, co-immunoprecipitation experiments confirmed the previously reported interaction of the BCA3 C-terminus with the catalytic subunit of PKAc [13]. Interestingly, PKAc also has been identified within highly purified HIV-1 particles [1]. We confirmed that PKAc is associated with HIV-1 virions regardless of the presence of BCA3. Finally, using a TZMb1 assay, we found that BCA3 incorporation has no impact on HIV-1 (pNL4-3) infectivity. Taken together, our data indicate that (i) full-length BCA3, but not its C-terminally truncated form, is packaged into HIV-1 particles; (ii) BCA3 is through its C-terminus able to bind to PKAc, which is also incorporated into HIV-1; (iii) the presence of BCA3 does not affect HIV-1 infectivity; (iv) knock-down of BCA3 in the virus-producing cells has no effect on the virus release; and (v) no HIV-1 region responsible for BCA3 binding and packaging into HIV-1 was identified. These findings suggest that BCA3 may be packaged into HIV-1 due to its ability to interact with PKAc.

The fact that BCA3 does not encode any specific structural/functional domain and has no homology to other known proteins complicates the prediction of its cellular function. Moreover, the data published about this protein to date are somewhat contradictory. BCA3 has been found to interact with functionally distinct proteins with different subcellular localizations, including the nucleus, cytosol and mitochondria. The proteins that have been identified to interact with or be affected by BCA3 include PKAc [4], NFκB [6,20], Rac [21], p73 [7], retroviral proteases [11,12], apoptosis-inducing factor (AIF) [10], apoptin [22], AKT [23] and ATP synthase [9]. Based on the cellular processes in which these BCA3-interacting partners participate, a broad spectrum of BCA3 activities has been suggested. These include functioning as a PKAc adaptor [4], upregulating NFκB-driven transcription in a PKAc-dependent manner [6], downregulating NFκB-driven transcription in a NEDDylation-dependent manner [5], serving as a molecular regulator for stress adaptation in the heart [10], serving as a stabilizer of MPT during myocardial ischaemia/reperfusion (I/R) injury [9], or inducing apoptosis [7,12]. From this list, it is clear that BCA3 function may vary depending on the cell type or clinical situation.

In this work, we analyzed the interaction of BCA3 with the catalytic subunit of PKAc. Sastri et al. showed that overexpression of BCA3 facilitates translocation and retention of endogenous PKAc in the nucleus, and they identified an NLS (R^14^, R^15^) at the BCA3 N-terminus [4]. A functional study of the PKAc–BCA3 interaction revealed that BCA3 shuttles PKAc into the nucleus, where the complex dissociates [13]. The potential interaction of NFκB p65 protein with the PKAc–BCA3 complex has also been suggested [6,13,20]. King et al. used a series of 15-mer peptides encompassing the entire BCA3 amino acid sequence to map the BCA3 binding site for PKAc, and found that the C-terminus of BCA3, specifically amino acids 150–179, comprises the PKAc binding site [13]. Our co-immunoprecipitation analysis confirmed that full-length BCA3, but not a C-terminally truncated form (Δ6BCA3), interacts with PKAc. Moreover, the deletion of the BCA3 C-terminus relocalized Δ6BCA3 out of the nucleus [4]. As BCA3 has been shown to facilitate translocation of PKAc into the nucleus, it is plausible that loss of its ability to bind PKAc by deletion of the C-terminus prevented Δ6BCA3 from entering the nucleus. This hypothesis is consistent with our finding that Δ6BCA3 is localized in the mitochondria even though it contains an intact NLS.

However, full-length BCA3 also was localized in the mitochondria. We have previously demonstrated that co-expression of BCA3 and HIV-1 PR in HEK 293 and HeLa cells leads to their mitochondrial localization [12]. Similarly, co-expression of BCA3 and p73 results in an association in the mitochondria [7]. Endogenously expressed BCA3 has been found in mitochondrial and nuclear fractions isolated from rat hearts [10], and BCA3 is enriched in cardiac mitochondria in mice with cardiac-specific overexpression of BCA3 [9]. The role of mitochondrially localized BCA3 has been connected with induction of apoptosis [7,12] and with direct interaction of BCA3 either with AIF, which is required for cell survival and mitochondrial integrity [10], or with ATP synthase, a key component of the MPT [9].

PKAc packaged within HIV-1 particles [1] is enzymatically active and able to phosphorylate a synthetic substrate in vitro [1]. A subsequent study showed that producing HIV-1 NL4.3 virus in the presence of a PKA inhibitor (H89) greatly affects the viral infectivity [2]. A detailed analysis revealed that HIV-1-associated PKAc acts as a cofactor that is required for early reverse transcription of the retroviral genome [2]. Our data confirmed that the BCA3 C-terminus is the binding site for PKAc, and that deletion of this region abolished both the Δ6BCA3–PKAc interaction and Δ6BCA3 incorporation into HIV-1 particles. In addition, we were unable to identify a BCA3 binding domain within the HIV-1 sequence. We also did not detect any impact of BCA3 on HIV-1 infectivity in a single-round infectivity assay. Based on these observations, we suggest that BCA3 incorporation into HIV-1 particles is mediated by its ability to interact with PKAc. BCA3 may be incorporated into HIV-1 merely as a PKAc-binding partner, which facilitates PKAc translocation into the nucleus; however, its role in the HIV-1 replication cycle remains uncertain. Alternatively, BCA3 may play a role during HIV-1 infection, but this is difficult to ascertain based on the current limited knowledge of BCA3 cellular function. Previously, we showed that HIV-1 PR is associated with BCA3 in mitochondria and that BCA3 increases the transcriptional activity of p53 on the *bax* promoter, which subsequently enhances apoptosis induced by HIV-1 [12]. Increased levels of p53 and bax following changes on the mitochondrial membrane also have been reported for HIV-1 infection of CD4+ T-cells [24]. It is therefore possible that the role of BCA3 may become evident during persistent HIV-1 infection.
